# β-Lactoglobulin Elevates Insulin and Glucagon Concentrations Compared with Whey Protein—A Randomized Double-Blinded Crossover Trial in Patients with Type Two Diabetes Mellitus

**DOI:** 10.3390/nu13020308

**Published:** 2021-01-22

**Authors:** Stine B. Smedegaard, Maike Mose, Adam Hulman, Ulla R. Mikkelsen, Niels Møller, Gregers Wegener, Niels Jessen, Nikolaj Rittig

**Affiliations:** 1 Steno Diabetes Center Aarhus, Aarhus University Hospital, Hedeager 3, 8200 Aarhus, Denmark; maikemose@clin.au.dk (M.M.); adahul@rm.dk (A.H.); niels.jessen@biomed.au.dk (N.J.); nikolaj.rittig@clin.au.dk (N.R.); 2 Arla Foods Ingredients Group P/S, Soenderhoej 10, 8260 Viby, Denmark; ulrmk@arlafoods.com; 3 Department of Diabetes and Hormone Diseases, Aarhus University Hospital, Palle Juul-Jensens Blv. 99, 8200 Aarhus, Denmark; niels.moeller@clin.au.dk; 4 Translational Neuropsychiatry Unit, Department of Clinical Medicine, Aarhus University, Noerrebrogade 44, Entrance 2B, 8000 Aarhus, Denmark; wegener@clin.au.dk; 5 Centre of Excellence for Pharmaceutical Sciences, North-West University, 11 Hoffman Street, Potchefstroom 2531, South Africa

**Keywords:** type 2 diabetes mellitus, whey, glucose, glycemic variability, beta-lactoglobulin, pre-meal, CGM

## Abstract

Whey protein is an insulinotropic fraction of dairy that reduces postprandial glucose levels in patients with type 2 diabetes mellitus (T2DM). We have recently shown that β-lactoglobulin (BLG), the largest protein fraction of whey, elevates insulin concentrations compared with iso-nitrogenous whey protein isolate (WPI) in healthy individuals. We therefore hypothesized that BLG pre-meals would lower glucose levels compared with WPI in patients with T2DM. We investigated 16 participants with T2DM using a randomized double-blinded cross-over design with two pre-meal interventions, (i) 25 g BLG and (ii) 25 g WPI prior to an oral glucose tolerance test (OGTT), followed by four days of continuous glucose monitoring (CGM) at home. BLG increased concentrations of insulin with 10%, glucagon with 20%, and glucose with 10% compared with WPI after the OGTT (all *p* < 0.05). Both BLG and WPI reduced the interstitial fluid (ISF) glucose concentrations (using CGM) with 2 mM and lowered glycemic variability with 10–15%, compared with tap-water (*p* < 0.05), and WPI lowered the ISF glucose with 0.5 mM compared with BLG from 120 min and onwards (*p* < 0.05). In conclusion, BLG pre-meals resulted in higher insulin, glucagon, and glucose concentrations compared with WPI in participants with T2DM. Pre-meal servings of WPI remains the most potent protein in terms of lowering postprandial glucose excursions.

## 1. Introduction

Pre-meals of whey protein have shown promising effects on the subsequent glucose trajectories in both healthy participants and patients with type 2 diabetes mellitus (T2DM) [1,2]. Whey given 15–30 min before a meal mediates a rise in insulin concentration and results in lower postprandial blood glucose concentrations [1,3]. The underlying mechanisms behind the insulinotropic properties observed following whey protein consumption are complex and not fully understood. Whey is especially rich in the branched chained amino acid (BCCA), leucine, which has direct insulin stimulating effect on the beta cell of the pancreas [4]. Whey protein also increases the concentration of the incretin hormones glucose-dependent insulinotropic polypeptide (GIP) [1,5,6] and glucagon-like peptide-1 (GLP-1) [1,5,7], which are also known to stimulate insulin secretion. Data from mouse pancreatic islets suggests that the exposure to an amino acid mixture and GIP [4], rather than one specific amino acid, has the greatest insulinotropic effects on the beta-cell.

Milk protein consists of around 80% casein and 20% whey [8]. Whey protein consists of 50–60% β-lactoglobulin (BLG), 17% α-lactalbumin, 10% immunoglobulins, 5% albumin and other polypeptides [9]. Recent data from our group show that BLG increases the serum(s)-concentration of insulin 23% more than a regular iso-nitrogenous whey protein isolate (WPI) in individuals without prior health issues. This observation led us to the hypothesis that pre-meal servings of BLG would stimulate insulin secretion and lower glucose trajectories compared with WPI in patients with T2DM. A more potent protein may lower protein and excessive calorie intake and improve compliance in prolonged protein pre-meal treatment regimes. Therefore, we performed a randomized double-blinded cross-over trial to investigate the effects of BLG and WPI pre-meals in patients with T2DM.

## 2. Materials and Methods

### 2.1. Study Approval

The study complied with the Declaration of Helsinki and was approved by the regional research ethics committee (1-10-72-226-19), registered at ClinicalTrials.gov (NCT04166760), and applied to the regulations of the Danish Data Protection Agency. All participants gave their written informed consent before inclusion in the study.

### 2.2. Participants

Participants were eligible for inclusion if they had T2DM, were between 18 and 80 years old, had a BMI between 20 and 35 kg/m^2^, had hemoglobin (Hb)-A1c between 40 and 69 mmol/L, and C-peptide between 370 and 1200 pmol/L. Recruitment was performed through social media (Facebook) and local newspapers. Exclusion criteria were milk allergies, daily intake of protein supplements, anti-glycemic medication other than metformin, or inability to speak or understand Danish. All participants were screened with a blood test panel of HbA1c, creatinine, thyrotropin, C-reactive-protein, sodium, potassium, albumin, alanine aminotransferase, alkaline phosphatase, bilirubin, hemoglobin, and C-peptide before inclusion.

### 2.3. Design and Protocol

**Figure 1:**
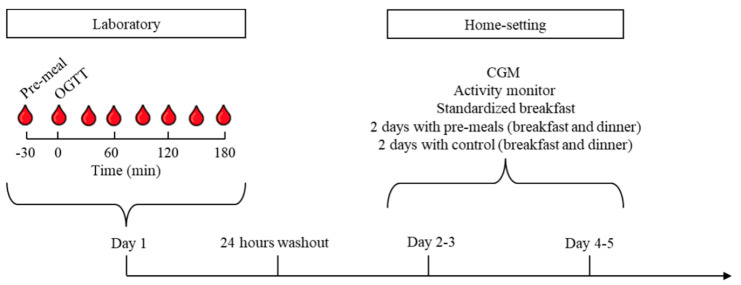
Flowchart of the study investigations. Participants were randomized to consume one of two pre-meals, (i) β-lactoglobulin (BLG) or (ii) whey protein isolate (WPI), 30 min before a 75 g oral glucose tolerance test (OGTT) in our laboratory or before breakfast and dinner at home. Participants were equipped with a continuous glucose monitor (CGM), an activity monitor, and standardized breakfast meals. Participants were also randomized to consume pre-meals before breakfast and dinner on days 2–3 or days 4–5 and control (tap-water) on the other two days. The experiment was repeated after 1 to 6 weeks from the OGTT.

The study was a randomized, double-blinded, cross-over trial with two interventions. Study days were identical except for interventions and consisted of an oral glucose tolerance test (OGTT) performed in our laboratory and four days of monitoring at home. The study was performed at the Steno/Medical laboratory, Aarhus University Hospital, Denmark. The two interventions consisted of: (i) BLG and (ii) whey protein isolate (WPI). There was a minimum washout period of one week and a maximum of six weeks between the two OGTTs. Participants and investigators were blinded in regard to the interventions. For an overview of the design and randomization, see Figure 1.

Before attending the laboratory, participants were asked to eat according to Danish nutritional guidelines (15% fat, 30% protein, and 55% carbohydrates) for 48 h and to avoid strenuous physical activity before and during each of the investigations (laboratory and home monitoring). If participants received metformin, this treatment was discontinued for five days before and during the investigations. All participants arrived following a 10-h overnight fast. During each study day, an intravenous catheter was placed in an antecubital vein for blood sampling. The participants consumed either 25 g of WPI or BLG 30 min before a 75 g OGTT was performed. Blood samples were collected consecutively in the three following hours.

Following the OGTT investigation, participants were equipped with a continuous glucose monitor (CGM), an activity monitor, four standardized breakfasts, a protein drink shaker, and four small plastic bags with 25 grams of the protein intervention. The first 24 h of CGM and activity recordings were used to calibrate equipment and excluded from analyses. Each participant was randomized to consume the protein pre-meals 30 min before the standardized breakfast and dinner during days two and three or during days four and five (Figure 1). Participants consumed an iso-voluminous amount of tap-water (CTR) 30 min before breakfast and dinner during the days without protein pre-meals. They were asked to avoid strenuous physical activity and eat similarly during the days of home-monitoring. The participants filled out a food-diary with timestamps for pre-meals and meals to ensure compliance and perform CGM analyses.

### 2.4. Interventions and Meals

The primary investigator enrolled and assigned participants to the sequence of interventions using www.randomizer.org [10]. The WPI (Lacprodan DI-9213) and BLG were provided by Arla Foods Ingredients Group P/S, Viby J, Denmark. The interventions were similar in appearance and taste. Two persons without relation to the investigations dosed and blinded 25 g of protein in small, labeled plastic bags. The proteins were dissolved in 200 mL of tap-water and served as a shake. The standardized breakfast consisted of 50 g cornflakes (Vores Cornflakes 500 g), 31 g raisins (Svansoe Rosiner 1500 g), and 250 mL skimmed milk (Arla^®^ Skummetmaelk 0.1% 250 mL) equivalent to 77.6 g carbohydrates, 13.9 g protein, 1 g fat/375 kCal. The characteristics of WPI and BLG are shown in Table 1. Participants and all persons involved in the trials, including the outcome assessors, remained blinded until statistical analyses had been performed. There were no adverse events reported.

### 2.5. Blood Analysis

Blood samples were drawn at −30, 0, 10, 20, 30, 40, 50, 60, 90, 120, 150, and 180 min following the OGTT. Plasma(p)-glucose was measured immediately using YSI 2300 model Stat Plus glucose analyzer (YSI Incorporated, Yellow Springs, OH). Blood for the remaining analyses was centrifuged at 4 °C, frozen at −20 °C, stored at −80 °C, and analyzed on the same assay after both arms of the study were completed for all participants. S-insulin, s-C-peptide, and p-glucagon concentrations were measured with an enzyme-linked immunosorbent assay (ELISA) technique using a commercial kit (Mercodia Insulin ELISA, Mercodia Glucagon ELISA, Mercodia C-peptide ELISA, Sweden). S-free fatty acids (FFA) were measured using the in vitro enzymatic colorimetric method assay NEFA-HR(2), which quantifies the concentration of non-esterified fatty acids (FUJIFILM Wako Chemicals Europe GmbH, Germany). P-amino acids (AA) concentrations were measured by high-pressure liquid chromatography (HPLC) method using a Thermo Scientific Ultimate 3000 system, as earlier described [11]. Briefly, the samples were diluted 1.11x by adding 2 M Perchloric acid (HCIO_4_) and then centrifuged at 14,000× *g* at 4 °C for 10 min. The supernatant was removed and filtered through a spin filter (0.22 µm) at 14,000× *g* for 1 min; then diluted 50× with 0.2 M HCIO_4_ to a final dilution factor of 55.5. Hereafter, the samples were injected into the HPLC. For separation, a Kinetex EVO C18 2.6 µm 4.6 × 150 mm column from Phenomenex, U.S., was used. Detection was done by fluorometric detection with excitation on 337 nm and emission on 442 nm. Samples for p-GIP and p-GLP-1 were extracted in final concentrations of 70% ethanol before analyses. Samples were analyzed on radioimmunoassays using antiserum #89390 for GLP-1 and antiserum #80867 for GIP targeting the C-terminal end of the hormones reacting equally with the intact hormone and the primary metabolites (N-terminally truncated) [12,13].

### 2.6. Continuous Glucose Monitoring

Continuous measurement of glucose concentrations in the interstitial fluid (ISF) was performed using a CGM device (NordicInfu Care Denmark, Dexcom G6, Dexcom Inc., San Diego, CA, USA). The device measures glucose every five minutes via a subcutaneous sensor. The participants wore the device on the abdomen and were unaware of their glucose level as the receiver was blinded. Data were uploaded to and analyzed in the software CLARITY (Dexcom CLARITY, v3.32.0, Dexcom Inc., San Diego, CA, USA). The mean glucose ± standard deviation (SD), daily maximum glucose level, and the coefficient of variation (CV) was used as outcome measures.

### 2.7. Activity Monitoring

A combined accelerometer and heart rate (HR) monitor (Actiheart 5 (AH), CamNtech Limited, Cambridge, UK) was used to evaluate the activity and estimate energy expenditure during the home investigation period. The AH unit was worn on and connected to the chest with two self-adhesive electrodes—one below the sternum and one under the left pectoral muscle. Data on accelerometry was collected at 32 Hz, and HR was collected as inter-beat-intervals. Data from the unit was uploaded to and analyzed using AH software (Actiheart software, version 5.1.10, camNtech ltd., Cambridge, UK). The software, processing of data, and validation of the system have been described in detail elsewhere [14]. Briefly, the AH software has a built-in function to correct missing beats and clean noise from the HR data. The software uses the cleaned HR data and data on activity in the integrated branched chained model “Group Cal JAP 2007” to estimate activity energy expenditure (AEE). In the case of missing HR data >5 min, the AEE is solely based on activity. The software provides the total energy expenditure (TEE) from a model using weight, height, age, sleeping heart rate (resting heart rate—10), AEE, and diet-induced thermogenesis. The software provides variables on HR, maximum HR, activity counts, AEE, and TEE.

### 2.8. Statistical Analysis

Statistical analyses and figures were conducted using the nlme (version 3.1-142), Epi (version 2.37) packages in R (R Foundation for Statistical Computing, Vienna, Austria, version 3.6.2) and SigmaPlot (San Jose, CA, USA, version 14.0). Trajectories on substrates and hormones in relation to the OGTT were fitted using random-effects models with a natural cubic spline specification for time. The number and position of the knots are different for those outcomes measured at a different set of time points. Interaction terms were included for each time term and a binary variable coding the two interventions. This, in combination with appropriate contrast matrices, allowed us to estimate trajectories for both interventions and their difference at any time point during the investigation. The differences between trajectories were expressed as percentages, as the outcomes were log-transformed (natural logarithm) before running the models due to their skewed distributions. Individual specific random intercepts and slopes were included in the models to account for the dependence within the data due to its repeated measurement nature. The same method was used to assess glucose trajectories during the three hours following breakfast and dinner for BLG, WPI, and CTR. For this analysis, measurements were included if their time points were after, but within three hours of, the recorded time of breakfast and dinner. The incremental area under the curve (iAUC) was calculated using the trapezoidal approach [15], and a paired t-test or one-way RM ANOVA was used for comparison of each outcome.

CGM-based summary measures and activity characteristics were compared between interventions and controls using random effects models with individual specific random intercepts to account for the cross-over design of the experiment. Differences between groups and their 95% confidence intervals (CIs) were estimated using the appropriate contrast matrices.

A pre-study power calculation with a significance level of 0.05 and a power of 80% was performed. We expected to detect a 25% difference in iAUC in insulin concentration (which was the primary outcome) between BLG and WPI with a 23% SD following the OGTT. This resulted in a sample size of 14. We expected a dropout rate of 10% and therefore included 16 participants.

## 3. Results

### 3.1. Participants

**Figure 2:**
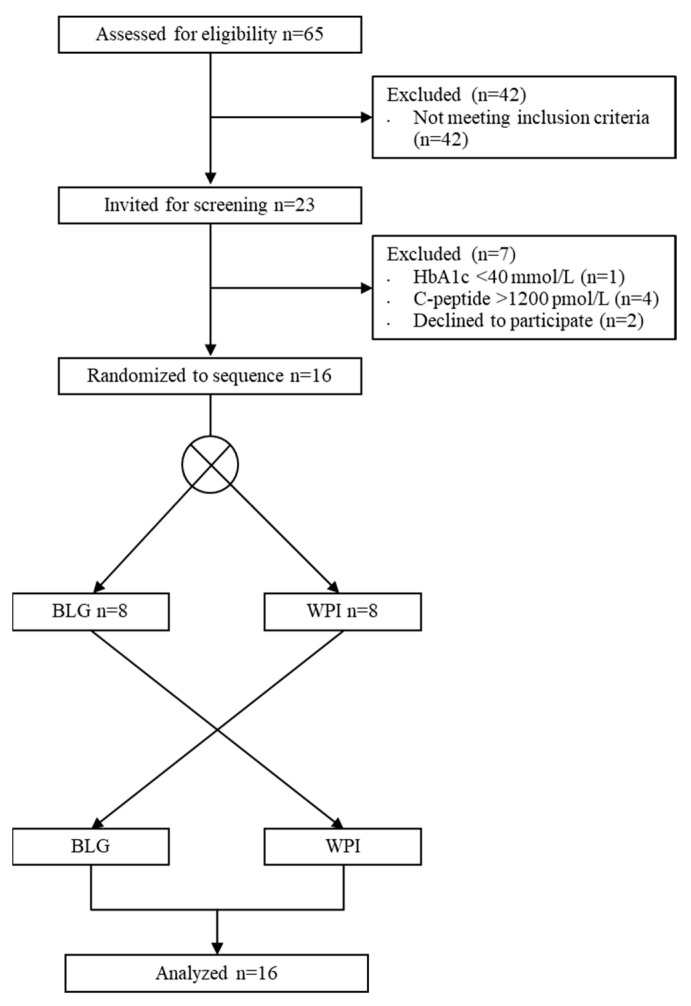
Flow diagram of inclusion in the randomized cross-over trial. WPI, whey protein isolate; BLG, β-lactoglobulin.

Sixty-five individuals were initially screened by the primary investigator over the phone (Figure 2). Sixteen participants were included and completed the studies between January 2020 and June 2020. One participant was unable to complete the home monitoring program. Patient characteristics are shown in (Table 2). There was a median washout-period of 9 days (range 7–23 days) between laboratory investigations.

### 3.2. Oral Glucose Tolerance Test (OGTT)

#### 3.2.1. Substrate and Hormone Concentrations

**Figure 3:**
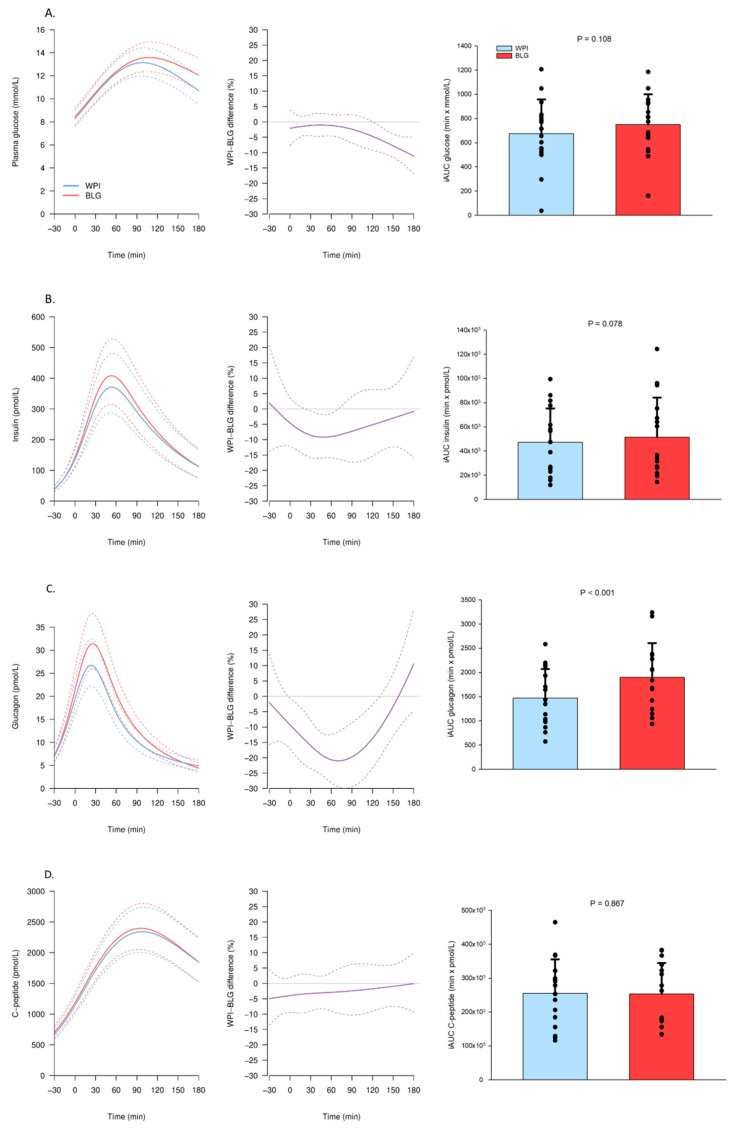
Plasma (p) and serum (s) concentrations of hormones and substrates after β-lactoglobulin (BLG) and whey protein isolate (WPI) pre-meals 30 min before an OGTT (0 min). Panels to the left show trajectories of the mean concentration (solid lines) with 95% confidence intervals (95% CIs) (dashed lines) of (**A**) p-glucose, (**B**) s-insulin, (**C**) p-glucagon, (**D**) s-C-peptide after WPI (blue) and BLG (red) consumption. The mean relative difference (solid line, purple) with 95% CIs (dashed lines) between the two interventions is shown in the middle panels. Panels to the right show the individual incremental area under the curve (iAUC) with a bar plot showing the mean ± standard deviation after WPI (blue) and BLG (red) consumption. *n* = 16.

**Figure 4:**
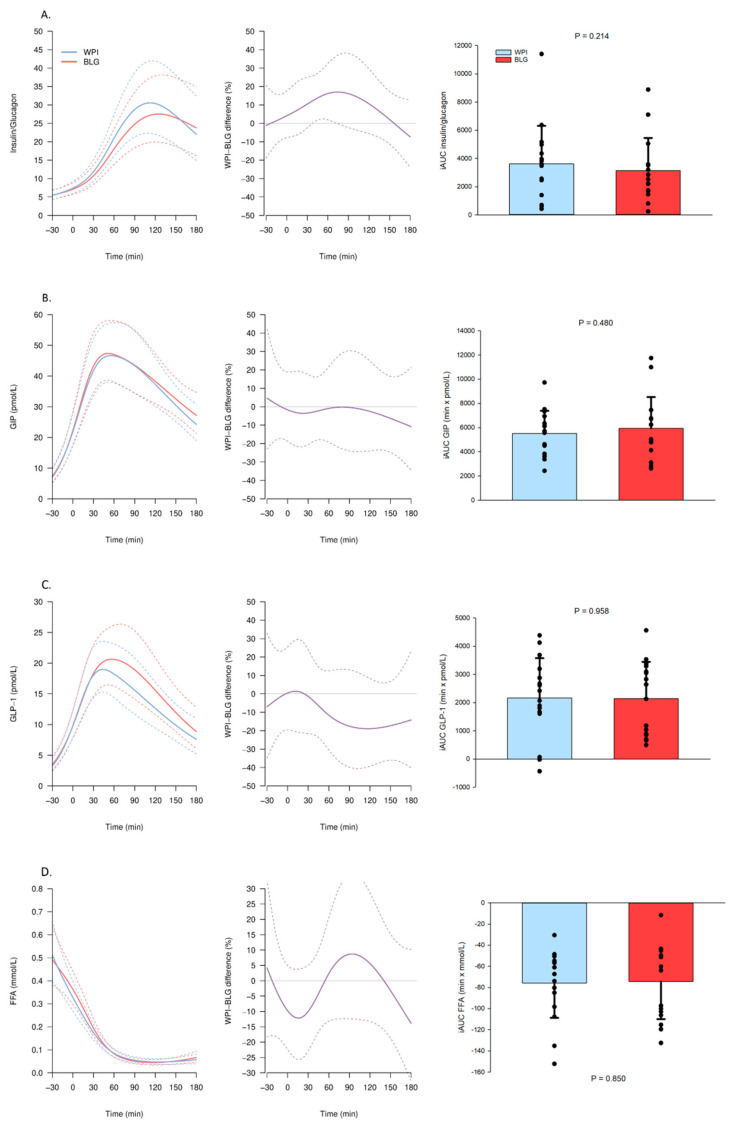
Plasma (p) and serum (s) concentrations of hormones and substrates after β-lactoglobulin (BLG) and whey protein isolate (WPI) pre-meals 30 min before an OGTT (0 min). Panels to the left show trajectories of the mean concentration (solid lines) with 95% confidence intervals (95% CIs) (dashed lines) of (**A**) s-insulin/p-glucagon ratio, (**B**) p-glucose-dependent insulinotropic polypeptide (GIP), (**C**) p-glucagon-like peptide-1 (GLP-1), (**D**) s-free fatty acids (FFA) after WPI (blue) and BLG (red) consumption. The mean relative difference (solid line, purple) with 95% CIs (dashed lines) between the two interventions is shown in the middle panels. Panels to the right show the individual incremental area under the curve (iAUC) with a bar plot showing the mean ± standard deviation after WPI (blue) and BLG (red) consumption. *n* = 16.

The p-glucose concentration was higher 120 min following BLG compared with WPI ingestion and reached a maximum difference of 10% 180 min following the OGTT (Figure 3A). The s-insulin concentration was elevated with 10% at 30–60 min and p-glucagon with 20% at 60–90 min after the OGTT following BLG compared with WPI (Figure 3B,C). Both BLG and WPI elevated s-C-peptide concentrations with no difference between BLG and WPI (Figure 3D). The WPI led to a higher insulin/glucagon ratio at 60 min (Figure 4A). Both proteins elevated p-GIP and p-GLP-1 concentrations and suppressed s-FFA concentrations with no difference between BLG and WPI (Figure 4B–D).

#### 3.2.2. Amino Acids

BLG elevated the p-concentration of aspartate, glutamate, leucine, lysine, methionine, phenylalanine, proline, and tyrosine compared with WPI (Figure S1). WPI elevated the p-concentration of glycine, isoleucine, serine, and threonine compared with BLG (Figure S1).

### 3.3. Home-Monitoring with Continuous Glucose Monitoring (CGM)

#### 3.3.1. CGM Glucose Trajectories Following Breakfast

**Figure 5:**
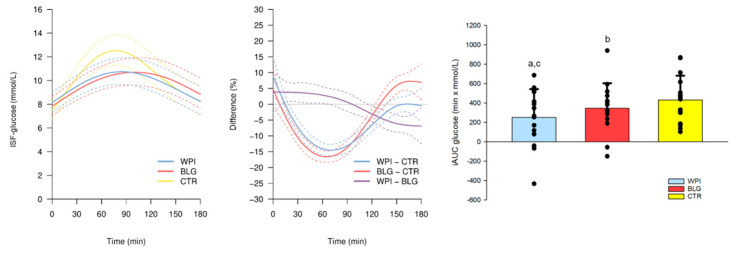
Interstitial fluid concentration of glucose (ISF-glucose) in the 180 min following intake of the pre-meals β-lactoglobulin (BLG), whey protein isolate (WPI), and control: tap-water (CTR) (−30 min) and standardized breakfast at home (0 min). The panel to the left shows trajectories of the mean ISF-glucose (solid lines) with 95% confidence intervals (95% CIs) (dashed lines) after WPI (blue), BLG (red) and CTR (yellow) consumption. The mean relative differences (solid lines) with 95% CIs (dashed lines) between the WPI and CTR (blue), BLG and CTR (red) and WPI and BLG (purple) are shown in the middle panel. The panel to the right shows the individual incremental area under the curve (iAUC) with a bar plot showing the mean ± standard deviation after WPI (blue), BLG (red) and CTR (yellow) consumption. One-way repeated measure ANOVA, *p* = 0.002, and post hoc (Student–Newman–Keuls) paired *t*-tests: a, WPI vs. CTR: *p* = 0.002; b, BLG vs. CTR: *p* = 0.077; c, WPI vs. BLG: *p* = 0.052. *n* = 15.

Both protein pre-meals lowered postprandial ISF-glucose concentration following breakfast with the largest difference of 15% (WPI) and 17% (BLG) (2 mM) around 60 min compared with CTR (Figure 5). In alignment with our results from the OGTT, the ISF-glucose was 7% (0.5 mM) lower after 150 min following WPI compared with BLG, but 4% higher around breakfast consumption (Figure 5).

#### 3.3.2. CGM and Summary Statistics

There was no difference in mean ISF-glucose between BLG, WPI, and CTR. The glycemic variability expressed as the CV was lower by 10% during WPI and by 15% during BLG, and the SD was lower by 9% during WPI and by 13% during BLG, compared with CTR. Additionally, after breakfast, the maximum glucose concentration was lower by 13% during WPI and 12% during BLG compared with CTR. The daily maximum glucose level was lower by 7% during WPI and 5% during BLG compared with CTR. No statistically significant differences were detected between BLG and WPI in any of the CGM summary variables (Table 3).

### 3.4. Energy Expenditure

TEE and AEE were higher on days with BLG compared with WPI. There was no significant difference between days with protein compared with CTR. Participants had similar activity counts, HR, and maximum HR on days with protein and days with CTR (Table 4).

## 4. Discussion

In this study, we showed how a BLG pre-meal served 30 min before an OGTT resulted in higher concentrations of insulin, glucagon, and glucose compared with WPI in patients with T2DM. The study was originally designed to investigate the insulinotropic properties of BLG with the hypothesis that elevated insulin concentrations would lower postprandial glucose excursions compared with WPI. We confirmed that BLG elevates insulin concentrations compared with WPI, but the simultaneous glucagonotropic effect also associated with BLG most likely explains why ISF-glucose concentrations were slightly higher (0.5 mM) following BLG compared with WPI, opposing our original hypothesis. Despite the similarity between the two dairy products, BLG contained more leucine and phenylalanine than WPI, which was also present in the p-concentrations of these specific AA following interventions. Both leucine and phenylalanine have been shown to stimulate insulin secretion [4,16] which, to some extent, may explain the insulinotropic properties. Also, p-concentrations of methionine and tyrosine have been shown to correlate with glucagon concentrations in humans [17], and perfusion studies in dogs and rodents have shown that aspartate, glutamate, lysine, and proline stimulate glucagon secretion [18,19]. These AA were all significantly higher after BLG consumption compared with WPI. Glucagon release is potently stimulated by GIP [20], but plasma concentrations of GIP following interventions were comparable between interventions. Hence, we suggest that the insulinotropic and glucagonotropic effects associated with BLG may relate to its specific AA composition.

Our study is the first to show glucose-lowering effect of pre-meal whey protein in a home-setting using CGM. Both interventions lowered glucose excursions with 2 mM following a standardized breakfast compared with tap-water (CTR). The effect is in line with other studies investigating similar doses of whey protein pre-meal servings [6,21]. To our knowledge, only one other study has investigated whey pre-meals in individuals with T2DM using CGM in a home-setting [22]. This study compared whey protein with a mixture of indigestible potato starch (carbohydrate rich) and could not show statistically significant effects on mean glucose levels, glucose trajectories, or glycemic variability following meals. It is well known that ingestion of small amounts of carbohydrates preceding a glucose load lowers the following glucose excursion, an effect referred to as the Staub-Traugott effect [23,24] that may explain why no significant differences were found in this study.

Only one study on long-term pre-meal whey exposure in T2DM has been performed (12 weeks) [21]. This study showed a small significant reduction in HbA1c (−1 mmol/mol). It should be noted that the participants were already well regulated with an HbA1c of 49 mmol/mol, which may have affected the size of the outcome. However, HbA1c does not necessarily reflect postprandial glucose excursions [25], and results might have been more substantial on glycemic variability. Large glucose excursions and high glycemic variability have been associated with risk of cardiovascular disease [26] as well as impaired cognitive function [27]. This emphasizes that minimizing postprandial glucose excursions may be important in the management of T2DM. We showed a reduction in glycemic variability, maximum glucose levels, and lower glucose excursions after consuming the pre-meal proteins compared with tap-water. Future long-term studies on pre-meal whey protein in participants with T2DM should preferably include investigations on glycemic variability.

Our study was limited, as the CTR intervention (tap-water) was unblinded. However, morning glucose concentrations were comparable between conditions, the participants were given the same standardized breakfast meal, and activity levels were similar between days with protein interventions and CTR. Activity and energy expenditure were comparable between groups, but showed a minor statistically significant elevation in TEE and AEE during BLG compared with WPI. These differences were small and only strengthen our findings showing lower glucose levels during WPI compared with BLG. We instructed participants to eat according to the Danish national recommendations (55% carbohydrates, 30% fat, and 15% protein) and to eat similar portion sizes during the home monitoring period. Still, the food diaries were generally of poor quality and lacked information. We did not include a control condition (e.g., tap-water) in the OGTT experiment because the primary aim of the study was to compare BLG and WPI. Pre-meals of whey have, in many previous studies, already proven effective in lowering glucose concentrations [2,3,21,28], but direct comparisons to other proteins are sparse.

A major strength of our study is the combination of investigations in a controlled laboratory and home setting. We included both men and women in our trial and both CGM and activity monitors in our investigations. The cross-over design eliminated any inter-individual differences.

In conclusion, a pre-meal of BLG elevates insulin, glucagon, and glucose concentration compared with WPI following an OGTT in patients with T2DM. Both WPI and BLG lowered glycemic variability and glucose trajectories compared with tap-water. WPI remains the most potent pre-meal in the management of postprandial glucose excursions.

## Figures and Tables

**Table 1:** Composition of the interventions; β-lactoglobulin (BLG) and whey protein isolate (WPI).

**Nutritional Content**	**BLG** **/100 g Product**	**WPI** **/100 g Product**
Total energy, kCal	375	355
Fat, g	0.1	0.1
Carbohydrate, g	0.1	0.1
Protein, g	93.5	88.3
No-calorie flavor, %	0.56	0.56
**Amino Acids**	**BLG** **g/100 g Protein**	**WPI** **g/100 g Protein**
Alanine	7.0	6.0
Arginine	2.8	2.3
Aspartic acid	12.1	11.8
Cysteine	3.1	2.6
Glutamic acid	20.1	19.7
Glycine	1.3	1.6
Histidine	1.7	1.7
Hydroxyproline	<0.1	<0.1
Isoleucine	6.3	7.0
Leucine	16.1	11.7
Lysine	12.3	10.6
Methionine	2.8	2.4
Ornithine	<0.1	<0.1
Phenylalanine	3.6	3.1
Proline	5.4	6.8
Serine	3.9	5.2
Threonine	5.3	8.0
Tryptophan	2.2	1.9
Tyrosine	3.7	2.9
Valine	6.1	6.4
Sum	115.9	111.8

The composition of amino acid analysis was done by Eurofins (GLP (Good Laboratory Practice) certified), and the nutritional content analysis was done by Danmark Protein (Arla Foods Ingredients).

**Table 2:** Demographic characteristics.

Characteristics	*n* = 16
Age, years	67.5 (40–78)
BMI, kg/m^2^	27.0 (21.2–32.9)
Women, %	56.2
Metformin treatment, *n*	14
HbA1c, mmol/mol	50 (43–55)
Fasting c-peptide, nmol/L	932 (49–1155)
Fasting insulin, pmol/L	40 (20–94)

Data are presented as absolute numbers or medians (ranges).

**Table 3:** Summary variables on continuous glucose monitoring.

***n* = 15**	**Mean Glucose, mmol/L**	**SD, mmol/L**	**CV, %**
CTR	8.7 (7.9; 9.5)	2.0 (1.8; 2.2)	22.7 (21.3; 24.1)
WPI	8.8 (8.0; 9.7)	1.8 (1.6; 2.0)	20.6 (18.9; 22.2)
BLG	8.6 (8.0; 9.7)	1.7 (1.5; 1.9)	19.2 (17.5; 20.9)
WPI–CTR	0.1 (−0.2; 0.4)	−0.2 (−0.3; −0.0) **	−2.2 (−3.9; −0.5) *
BLG–CTR	0.2 (−0.1; 0.5)	−0.3 (−0.4; −0.1) **	−3.5 (−5.2; −1.8) **
WPI–BLG	−0.1 (−0.4; 0.3)	0.1 (−0.1; 0.3)	1.3 (−0.6; 3.3)
***n* = 15**	**Max after Breakfast, mmol/L**	**Daily max, mmol/L**	**Max after Dinner, mmol/L**
CTR	14.4 (13.1; 15.6)	14.5 (13.2; 15.9)	10.8 (9.8; 11.8)
WPI	12.5 (11.2; 13.9)	13.5 (12.2; 14.9)	10.9 (9.7; 12.0)
BLG	12.7 (11.4; 14.0)	13.8 (12.5; 15.2)	11.0 (9.8; 12.1)
WPI–CTR	−1.8 (−2.4; −1.3) *	−1.0 (−1.6; −0.4) **	0.1 (−0.8; 1.0)
BLG–CTR	−1.7 (−2.2; −1.1) **	−0.7 (−1.3; −0.1) *	0.2 (−0.7; 1.1)
WPI–BLG	−0.2 (−0.8; 0.5)	−0.3 (−1.0; 0.4)	−0.1 (−1.1; 0.9)

Coefficient of variation (CV) and standard deviation (SD) as parameters on glycemic variability. Maximum (max) after breakfast and dinner is the peak in the postprandial glucose concentration during three hours following the meals. Values are presented as means with 95% confidence intervals. * *p* < 0.05, ** *p* < 0.01, statistically significant differences are highlighted (bold). CTR, control; WPI, whey protein isolate; BLG, beta-lactoglobulin.

**Table 4:** Activity measurements and energy expenditure.

*n* = 15	TEE, kCal	AEE, kCal	Activity, counts/min	Mean HR, BPM	Maximum HR, BPM
CTR	2446 (2234; 2658)	659 (514; 804)	34 (26; 41)	74 (70; 79)	104 (97; 110)
WPI	2338 (2110; 2565)	559 (396; 721)	28 (19; 36)	75 (70; 79)	103 (96; 111)
BLG	2499 (2277; 2722)	708 (551; 865)	34 (25; 42)	76 (71; 80)	105 (98; 112)
WPI–CTR	−109 (−243; 26)	−100 (−221; 20)	−7 (−13; 0) *	0 (−2; 3)	0 (−5; 5)
BLG–CTR	53 (−74; 180)	49 (−66; 163)	0 (−6; 6)	2 (−1; 4)	2 (−3; 7)
WPI–BLG	−161 (−313; −10) *	−149 (−286; −13) *	−6 (−14; 1)	−1 (−4; 1)	−2 (−8; 4)

Data are expressed as means with 95% confidence intervals. * *p* < 0.05, statistically significant differences are highlighted (bold). TEE, total energy expenditure; kCal, kilocalories; AEE, activity energy expenditure; HR, heart rate; BPM, beats per minute; CTR, control; WPI, whey protein isolate; BLG, beta-lactoglobulin.

## Data Availability

The data presented in this study are available on request from the corresponding author. The data are not publicly available due to privacy.

## Supplementary Materials

The following are available online at https://www.mdpi.com/2072-6643/13/2/308/s1, Figure S1: Plasma concentrations amino acids (AA) after β-lactoglobulin (BLG) and whey protein isolate (WPI) pre-meals 30 min before an OGTT (0 min). Panels to the left show trajectories of the mean concentration (solid lines) with 95% confidence intervals (95%CIs) (dashed lines) of (A) alanine, (B) arginine, (C) aspartate, (D) glutamate, (E) glycine, (F) histidine, (G) isoleucine, (H) leucine, (I) lysine, (J) methionine, (K) phenylalanine, (L) proline, (M) serine, (N) threonine, (O) tyrosine, (P) valine, (Q) total AA after WPI (blue) and BLG (red) consumption. The mean relative difference (solid line, purple) with 95%CIs (dashed lines) between the two interventions is shown in the middle panel. Panels to the right show the individual incremental area under the curve (iAUC) with a bar plot showing the mean ± standard deviation after WPI (blue) and BLG (red) consumption. N = 16.

## References

[1] Ma J., Stevens J.E., Cukier K., Maddox A.F., Wishart J.M., Jones K.L., Clifton P.M., Horowitz M., Rayner C.K. (2009). Effects of a protein preload on gastric emptying, glycemia, and gut hormones after a carbohydrate meal in diet-controlled type 2 diabetes. Diabetes Care.

[2] Akhavan T., Luhovyy B.L., Brown P.H., Cho C.E., Anderson G.H. (2010). Effect of premeal consumption of whey protein and its hydrolysate on food intake and postmeal glycemia and insulin responses in young adults. Am. J. Clin. Nutr..

[3] Bjørnshave A., Holst J.J., Hermansen K. (2018). Pre-Meal Effect of Whey Proteins on Metabolic Parameters in Subjects with and without Type 2 Diabetes: A Randomized, Crossover Trial. Nutrients.

[4] Salehi A., Gunnerud U., Muhammed S.J., Ostman E., Holst J.J., Björck I., Rorsman P. (2012). The insulinogenic effect of whey protein is partially mediated by a direct effect of amino acids and GIP on β-cells. Nutr. Metab..

[5] Nilsson M., Holst J.J., Björck I.M. (2007). Metabolic effects of amino acid mixtures and whey protein in healthy subjects: Studies using glucose-equivalent drinks. Am. J. Clin. Nutr..

[6] Frid A.H., Nilsson M., Holst J.J., Björck I.M. (2005). Effect of whey on blood glucose and insulin responses to composite breakfast and lunch meals in type 2 diabetic subjects. Am. J. Clin. Nutr..

[7] Jakubowicz D., Froy O., Ahrén B., Boaz M., Landau Z., Bar-Dayan Y., Ganz T., Barnea M., Wainstein J. (2014). Incretin, insulinotropic and glucose-lowering effects of whey protein pre-load in type 2 diabetes: A randomised clinical trial. Diabetologia.

[8] Zheng H., Clausen M.R., Dalsgaard T.K., Bertram H.C. (2015). Metabolomics to Explore Impact of Dairy Intake. Nutrients.

[9] Layman D.K., Lönnerdal B., Fernstrom J.D. (2018). Applications for α-lactalbumin in human nutrition. Nutr. Rev..

[10] Urbaniak G.C., Plous S. Research Randomizer (Version 4.0) [Computer Software]. http://www.randomizer.org/.

[11] Liebenberg N., Jensen E., Larsen E.R., Kousholt B.S., Pereira V.S., Fischer C.W., Wegener G. (2018). A Preclinical Study of Casein Glycomacropeptide as a Dietary Intervention for Acute Mania. Int. J. Neuropsychopharmacol..

[12] Orskov C., Rabenhøj L., Wettergren A., Kofod H., Holst J.J. (1994). Tissue and plasma concentrations of amidated and glycine-extended glucagon-like peptide I in humans. Diabetes.

[13] Lindgren O., Carr R.D., Deacon C.F., Holst J.J., Pacini G., Mari A., Ahrén B. (2011). Incretin hormone and insulin responses to oral versus intravenous lipid administration in humans. J. Clin. Endocrinol. Metab..

[14] CamNtech Ltd. (2020). Actiheart User Manual 5.1.14.

[15] Brouns F., Bjorck I., Frayn K.N., Gibbs A.L., Lang V., Slama G., Wolever T.M. (2005). Glycaemic index methodology. Nutr. Res. Rev..

[16] Van Loon L.J., Saris W.H., Verhagen H., Wagenmakers A.J. (2000). Plasma insulin responses after ingestion of different amino acid or protein mixtures with carbohydrate. Am. J. Clin. Nutr..

[17] Calbet J.A.L., MacLean D.A. (2002). Plasma Glucagon and Insulin Responses Depend on the Rate of Appearance of Amino Acids after Ingestion of Different Protein Solutions in Humans. J. Nutr..

[18] Rocha D.M., Faloona G.R., Unger R.H. (1972). Glucagon-stimulating activity of 20 amino acids in dogs. J. Clin. Investig..

[19] Galsgaard K.D., Jepsen S.L., Kjeldsen S.A.S., Pedersen J., Wewer Albrechtsen N.J., Holst J.J. (2020). Alanine, arginine, cysteine, and proline, but not glutamine, are substrates for, and acute mediators of, the liver-α-cell axis in female mice. Am. J. Physiol. Endocrinol. Metab..

[20] Lund A., Vilsbøll T., Bagger J.I., Holst J.J., Knop F.K. (2011). The separate and combined impact of the intestinal hormones, GIP, GLP-1, and GLP-2, on glucagon secretion in type 2 diabetes. Am. J. Physiol. Endocrinol. Metab..

[21] Watson L.E., Phillips L.K., Wu T., Bound M.J., Checklin H.L., Grivell J., Jones K.L., Clifton P.M., Horowitz M., Rayner C.K. (2019). A whey/guar "preload" improves postprandial glycaemia and glycated haemoglobin levels in type 2 diabetes: A 12-week, single-blind, randomized, placebo-controlled trial. Diabetes Obes. Metab..

[22] Almario R.U., Buchan W.M., Rocke D.M., Karakas S.E. (2017). Glucose-lowering effect of whey protein depends upon clinical characteristics of patients with type 2 diabetes. BMJ Open Diabetes Res. Care.

[23] Staub H. (1921). Untersuchungen über den Zuckerstoffwechsel des Menschen (Studies on sugar metabolism of humans). Z. Klin. Med..

[24] Traugott K. (2005). Über das Verhalten des Blutzuckerspiegels bei Wiederholter und Verschiedener Art Enteraler Zuckerzufuhr und Dessen Bedeutung für die Leberfunktion (About the behavior of blood sugar level in repeated and different types of enteral sugar intake and their impact on liverfunction). Klin. Wochenschr..

[25] Battelino T., Danne T., Bergenstal R.M., Amiel S.A., Beck R., Biester T., Bosi E., Buckingham B.A., Cefalu W.T., Close K.L. (2019). Clinical Targets for Continuous Glucose Monitoring Data Interpretation: Recommendations from the International Consensus on Time in Range. Diabetes Care.

[26] Cavalot F., Pagliarino A., Valle M., Di Martino L., Bonomo K., Massucco P., Anfossi G., Trovati M. (2011). Postprandial blood glucose predicts cardiovascular events and all-cause mortality in type 2 diabetes in a 14-year follow-up: Lessons from the San Luigi Gonzaga Diabetes Study. Diabetes Care.

[27] Xia W., Luo Y., Chen Y.C., Chen H., Ma J., Yin X. (2020). Glucose Fluctuations Are Linked to Disrupted Brain Functional Architecture and Cognitive Impairment. J. Alzheimer’s Dis..

[28] King D.G., Walker M., Campbell M.D., Breen L., Stevenson E.J., West D.J. (2018). A small dose of whey protein co-ingested with mixed-macronutrient breakfast and lunch meals improves postprandial glycemia and suppresses appetite in men with type 2 diabetes: A randomized controlled trial. Am. J. Clin. Nutr..

