# Supplementary material for: β-Lactoglobulin Elevates Insulin and Glucagon Concentrations Compared with Whey Protein—A Randomized Double-Blinded Crossover Trial in Patients with Type Two Diabetes Mellitus
Source: Nutrients. 2021 Jan 22;13(2):308. doi: 10.3390/nu13020308 (PMC7911205; doi:10.3390/nu13020308)
Supplement: Supplementary file 1 [file nutrients-13-00308-s001.pdf]

Supplementary Material

Figure S1. Amino Acids in Plasma

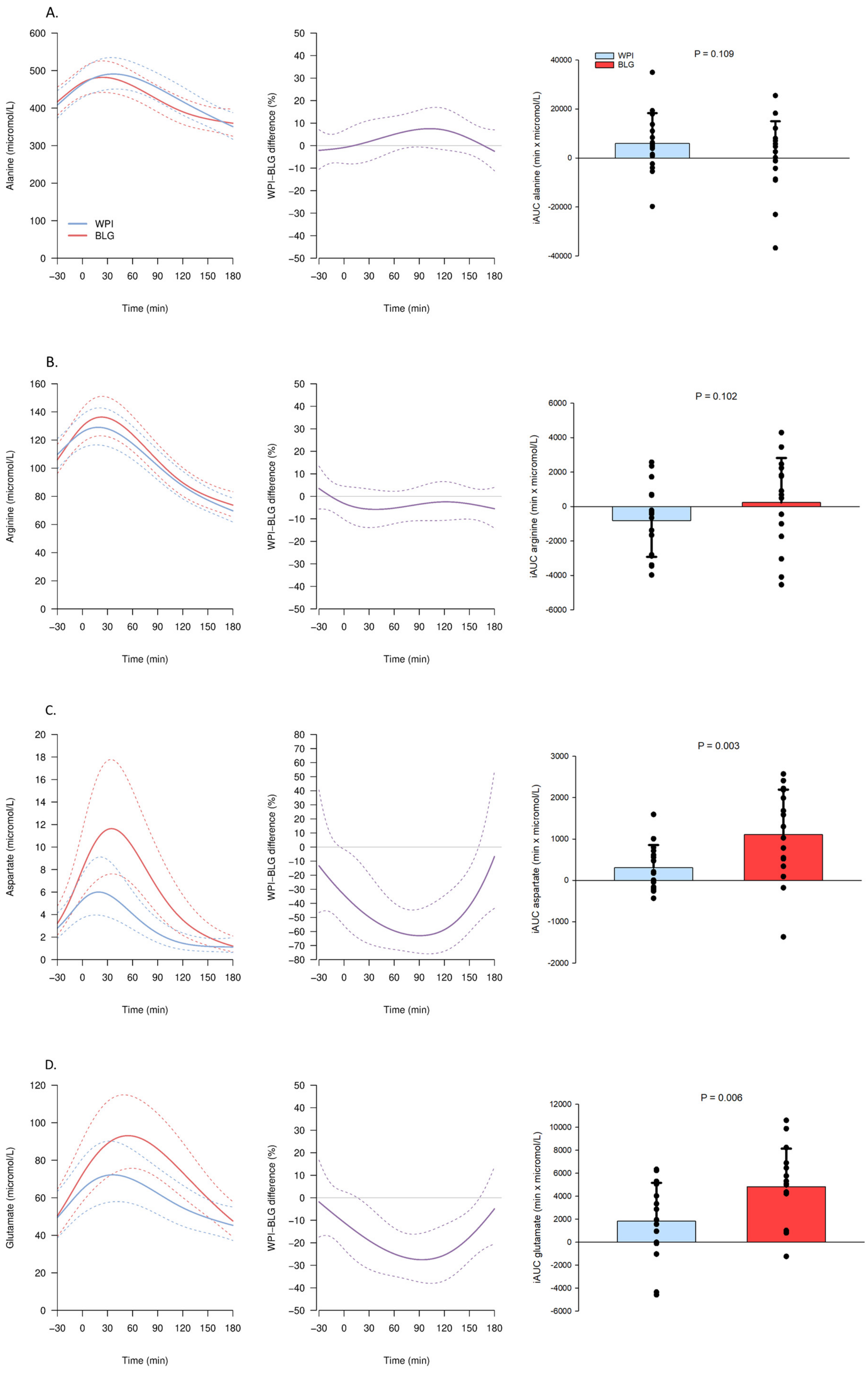

Supplementary Material

Figure S1. Amino Acids in Plasma

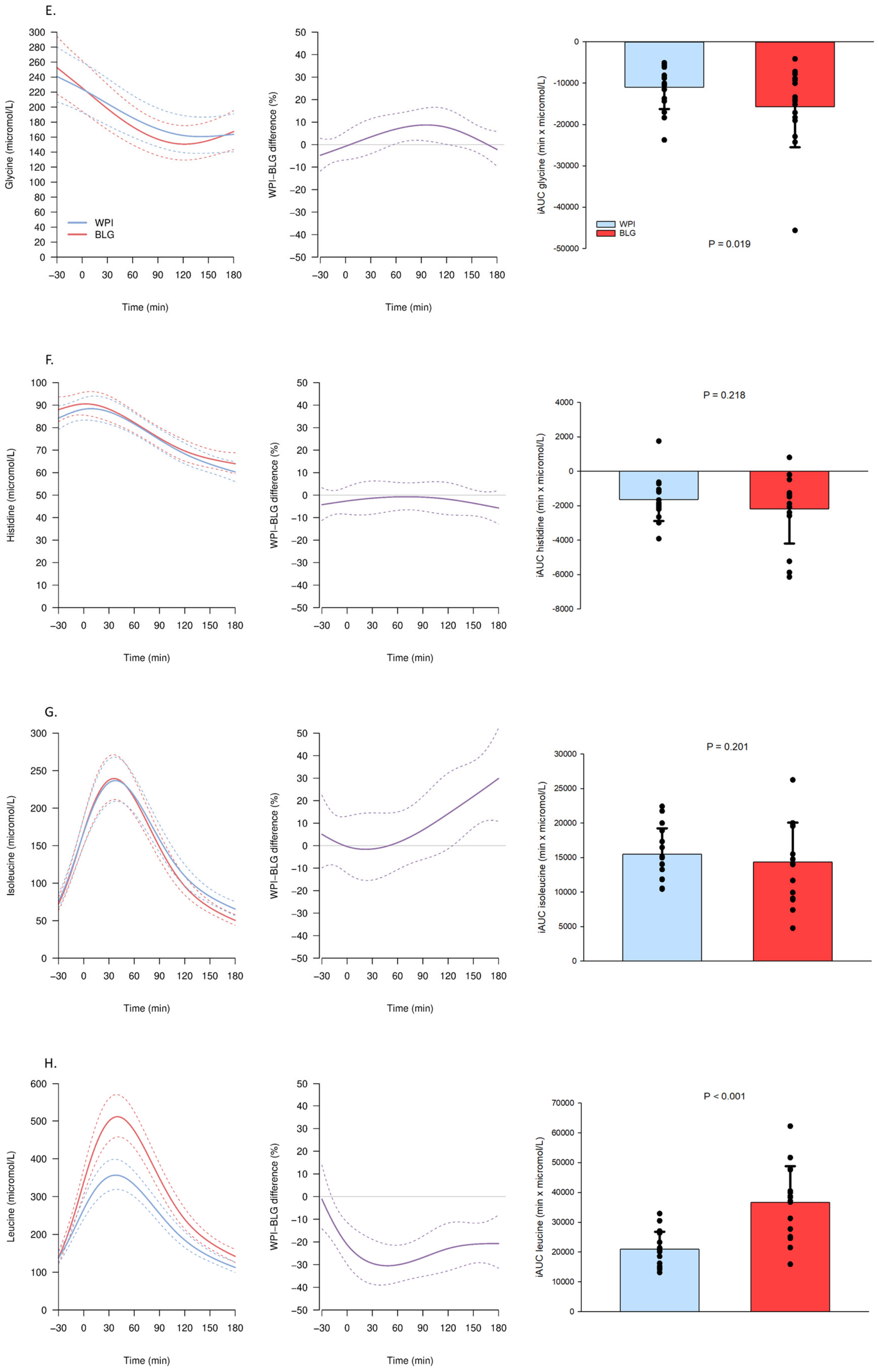

Supplementary Material

Figure S1. Amino Acids in Plasma

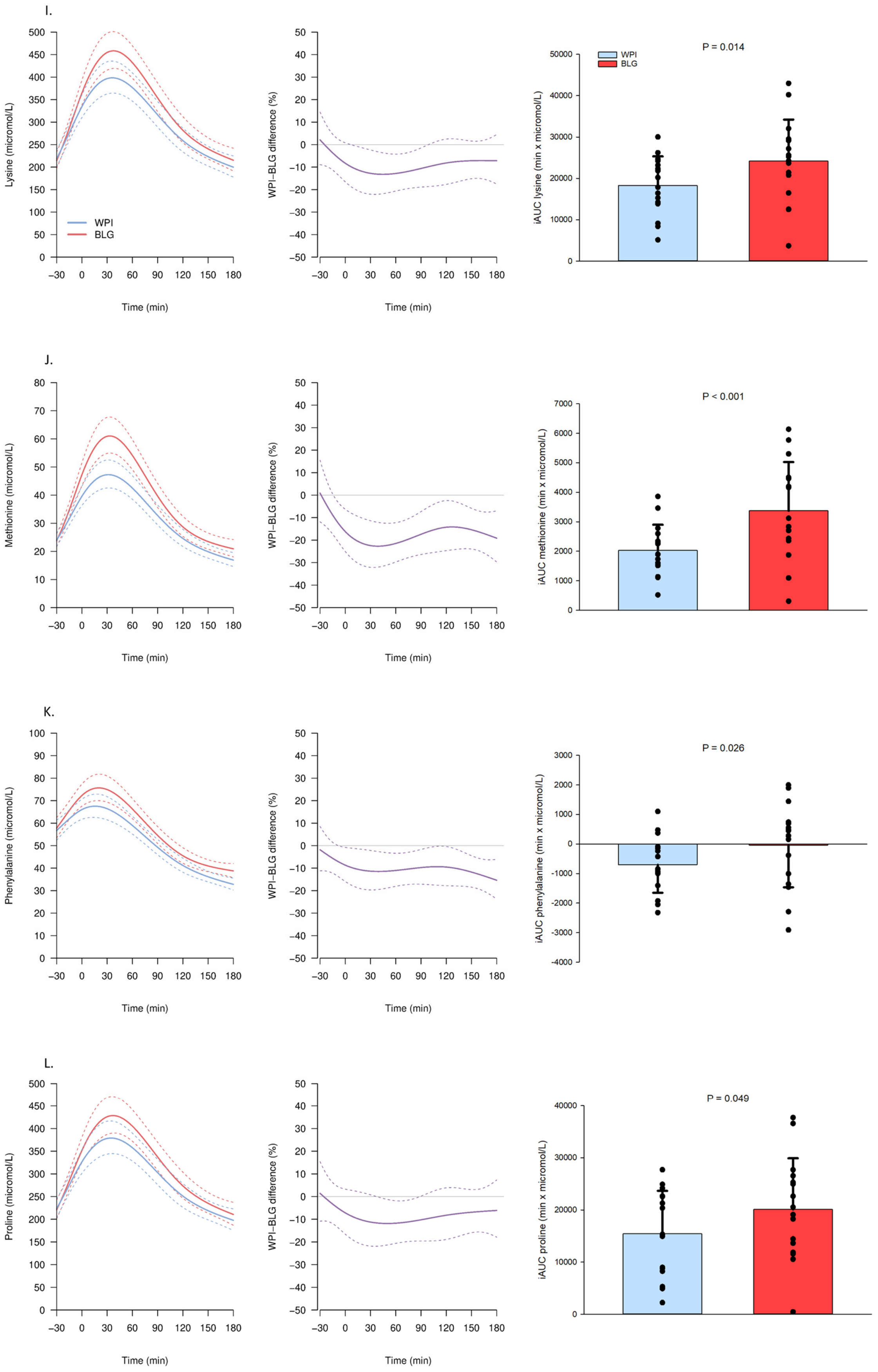

Supplementary Material

Figure S1. Amino Acids in Plasma

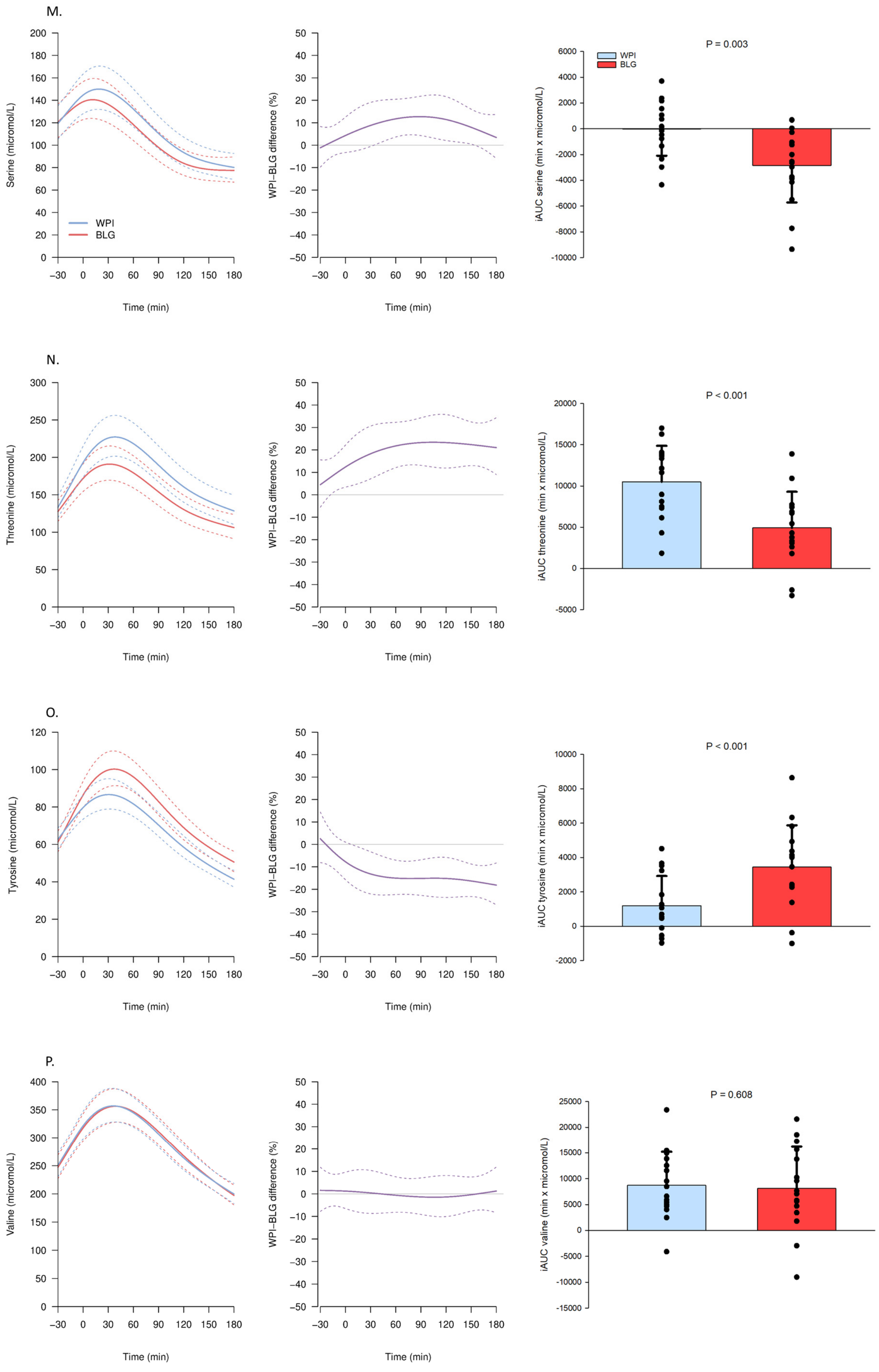

Supplementary Material

Figure S1. Amino Acids in Plasma

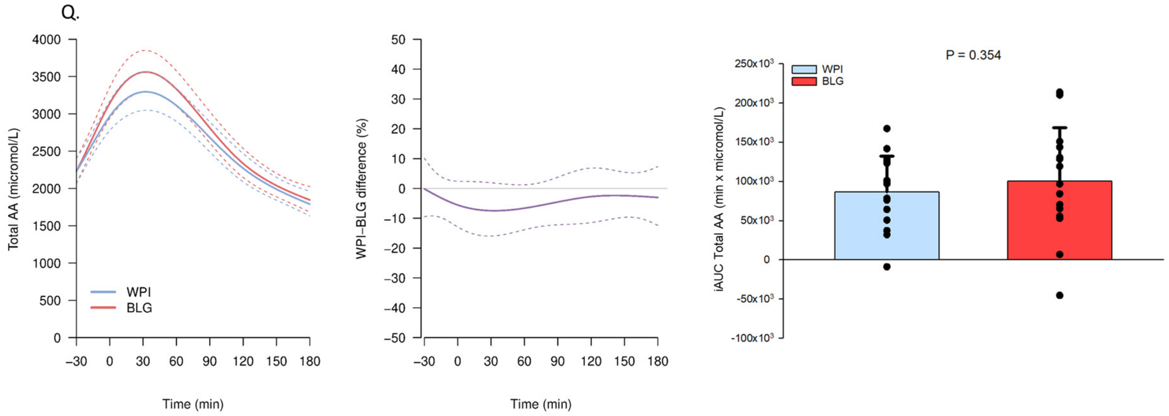

Figure S1. Plasma concentrations amino acids (AA) after β-lactoglobulin (BLG) and whey protein isolate (WPI) pre-meals 30 min before an OGTT (0 min). Panels to the left show trajectories of the mean concentration (solid lines) with 95% confidence intervals (95%CIs) (dashed lines) of A) alanine, B) arginine, C) aspartate, D) glutamate, E) glycine, F) histidine, G) isoleucine, H) leucine, I) lysine, J) methionine, K) phenylalanine, L) proline, M) serine, N) threonine, O) tyrosine, P) valine, Q) total AA after WPI (blue) and BLG (red) consumption. The mean relative difference (solid line, purple) with 95%CIs (dashed lines) between the two interventions is shown in the middle panel. Panels to the right show the individual incremental area under the curve (iAUC) with a bar plot showing the mean ± standard deviation after WPI (blue) and BLG (red) consumption. N=16.
